# Complete Genome Sequence of XaF13, a Novel Bacteriophage of Xanthomonas vesicatoria from Mexico

**DOI:** 10.1128/MRA.01371-19

**Published:** 2020-01-30

**Authors:** Guillermo Alejandro Solís-Sánchez, Evangelina Esmeralda Quiñones-Aguilar, Saul Fraire-Velázquez, Julio Vega-Arreguín, Gabriel Rincón-Enríquez

**Affiliations:** a Laboratorio de Fitopatología, Unidad de Biotecnología Vegetal, Centro de Investigación y Asistencia en Tecnología y Diseño del Estado de Jalisco, A.C. (CIATEJ) Zapopan, Jalisco, Mexico; b Laboratorio de Biología Integrativa de Plantas y Microorganismos, Unidad Académica de Ciencias Biológicas, Universidad Autónoma de Zacatecas (UAZ), Zacatecas, Zacatecas, Mexico; c ENES-León, Universidad Nacional Autónoma de México, León, Guanajuato, Mexico; Portland State University

## Abstract

The phytopathogenic bacterium Xanthomonas vesicatoria is the causative agent of bacterial spot disease in various Solanaceae family members. Here, we describe the complete genome sequence of XaF13, a novel filamentous phage that infects the phytopathogenic bacterium X. vesicatoria. The 7,045-bp genome is predicted to encode 14 open reading frames, 7 of which are related to those of other filamentous Xanthomonas phages.

## ANNOUNCEMENT

Mexico is the second most important producer of peppers worldwide. The production of peppers faces many phytosanitary problems such as bacterial spot disease (1–3). Bacterial spot disease control is difficult because Xanthomonas species can survive for long periods in seeds and crop debris (4). The use of bacteriophages against bacteria is considered a possible alternative to agrochemicals (5).

The bacteriophage XaF13 (family Inoviridae) was isolated from a soil sample collected in Yurécuaro, Mexico, from a pepper field affected with bacterial spot disease. A soil sample was suspended in peptone yeast glycerol (NYG) medium (100 ml) (6), inoculated with Xanthomonas vesicatoria (laboratory strain BV865), and incubated for 24 h at 28°C. The resulting slurry was centrifuged (8,000 × *g*; 20 min), and the supernatant was filtered (0.22 μm). Phages in the supernatant were isolated in double-layer plaque assays (0.7% agar) (7). Lysis plaques of ∼1 mm were observed in the bacterial lawn; then, a single plaque was isolated and purified two times.

DNA extraction from the bacteriophage (>10^10^ PFU/ml) was performed using a phage DNA extraction kit (Norgen-Biotek, Canada). The nature of the genome was characterized using DNase I (Roche, Germany), RNase A (Sigma-Aldrich, USA), and S1 nuclease (Promega, USA) according to the manufacturers’ instructions. The DNA of XaF13 was amplified using the Illustra TempliPhi amplification kit (GE Healthcare, UK); next, the DNA library was prepared with the Nextera XT DNA sample preparation kit (Illumina, USA), and the library quality was analyzed with a Bioanalyzer 2010 instrument (Agilent Technologies). High-throughput sequencing was performed by synthesis protocol (MiSeq; Illumina) with a 2 × 300-bp paired-end run at the sequencing laboratory at the Universidad Autónoma de Zacatecas (UAZ). The raw reads were filtered using Trimmomatic v. 0.38, the genome assembly was performed using SPAdes v. 3.11.0 (8), and the quality of the assembly was analyzed using QUAST v. 5.0.0 (9). To annotate the XaF13 genome, first, potential coding sequences were searched using BLAST-X; next, Pfam and HMMer were used to find homologues and conserved domains, and the best hits were grouped as a single locus. The specialized online tools PHANOTATE (10) and PHASTER (11) were used to predict specific phage genes, while tRNAs and rRNAs were predicted using tRNAscan-SE (12) and Rfam (13); transcriptional promoters were predicted using PromoterHunter (14). Default parameters were used for all software and tools except BLAST-X and PromoterHunter.

**FIG 1:**
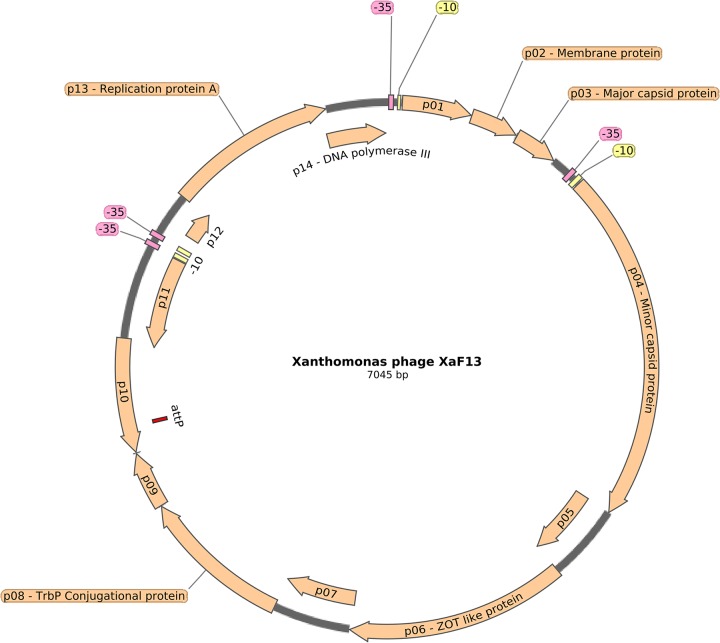
XaF13 phage genome organization. Arrows represent predicted genes and transcription direction. Promoter positions are indicated by yellow (−10) and pink (−35) boxes and the *attP* site by a red box. The illustration was prepared using SnapGene software (GSL Biotech).

A total of 394,491 reads were obtained, and the genome of XaF13 was assembled into a single contig with a median coverage of 33,597×. The genome is composed of a single-stranded DNA molecule, as shown by the DNase I, RNase A and S1 nuclease results, with a length of 7,045 bases and a G+C content of 60.3%. Bioinformatic analysis revealed 14 open reading frames (ORFs), 12 in the positive strand and 2 in the negative strand (replicative form); no tRNAs or rRNAs were found. Seven ORFs were annotated as hypothetical proteins, and 7 were annotated as having virus-related functions, namely, ORF-2 (membrane protein), ORF-3 (major capsid protein), ORF-4 (minor capsid protein), ORF-6 (ZOT-like protein), ORF-8 (TrbP conjugational protein), ORF-13 (replication protein A), and ORF-14 (DNA polymerase III). Moreover, an attachment site (15 bp) was identified by nucleotide comparison between XaF13 and *Xanthomonas* phage Xf109, similar to the *attP* sequences of lysogenic *Xanthomonas* phages Cf1c, XacF1, and φLf, which use the XerC/D recombinases from their hosts to integrate into the *dif* locus of the bacterial genome (15). Finally, regulatory sequences, located at positions −10 and −35, suggest that the XaF13 genome is organized into 4 functional regions, 3 of which are associated with structural functions, conjugation, and replication; the fourth is formed by ORFs 10 and 11, whose functions have not been defined so far (Fig. 1). The comparison of DNA sequences between the XaF13 and 40 Inoviridae phages using BLASTN showed that XaF13 is related to the filamentous phage Xf109 of Xanthomonas oryzae (57% query coverage and 86% nucleotide identity).

### Data availability.

The complete genome sequence of phage XaF13 is available in the GenBank database under accession number MN335248. The raw sequence reads are available in the SRA database under accession number SRX6866382 (BioProject number PRJNA566170).
